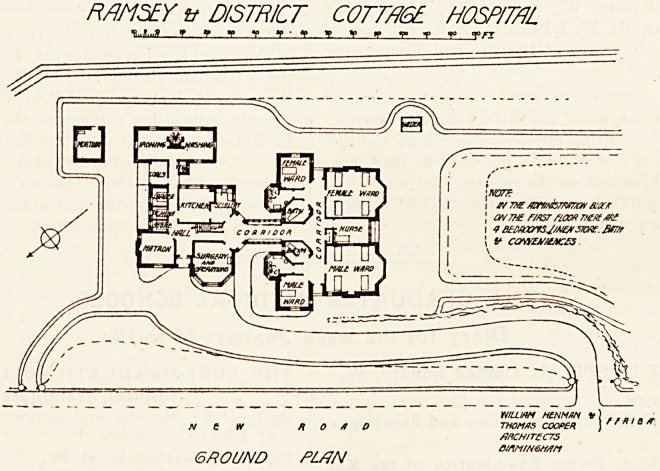# The Ramsey Cottage Hospital

**Published:** 1908-01-11

**Authors:** 


### THE RAMSEY COTTAGE HOSPITAL.

IT would not be right to apply with too much strictness the same canons of hospital construction to a small cottage hospital, as it would be to a large general infirmary. In the latter case the architect employed is more likely to have a free hand and sufficient money. In the former he is often cramped as regards both money and site. These facts are pointed out in the description sent to us of this building, and we fully admit their significance.

This hospital is very compact, and the positions of the various parts in relation to each other are nearly all good. In general design it bears considerable resemblance to the Watford Cottage Hospital, as given on page 264 of "Burdett's Cottage Hospitals." The front part is one story only, and it faces south-west. It consists of a nurses' room in the centre, and has on one side a male ward for four beds, and on the other a female ward for the same number of beds. Each ward has a large bay window in front and a small window at the side; and there is, therefore, a certain amount of cross ventilation obtainable; but we should like to have seen more done to effect this either by larger side windows or running out the ends of the wards some feet beyond their present position.

Behind these wards and nurses' room is a corridor and at each end of the corridor is an entrance. This is much better than having the entrance in the front elevation as it is at Watford. Near these entrances are the single-bedded wards, which take the place of the sanitary blocks at Watford; and here, again, the Ramsey plan is an improvement. Close to the single-bedded wards are the bath-rooms and closets. The former will pass muster in so small a hospital; but the latter should certainly have been pushed out far enough to obtain a cross-ventilated lobby.

At right-angles to the corridor already mentioned is another corridor which is well lighted, and which connects the kitchen department with the hospital proper. The surgery, which is also the operating-room, is, however, placed in this department, and quite correctly it has a northern aspect. The matron's room is at the north corner, and between this and the surgery is another entrance. The laundry is connected by a covered way with the kitchen department. This part has a first floor on which are four bedrooms, store-room, bath-room, and closet. The mortuary is entirely separate.

RAMSEY & DISTRICT COTTAGE HOSPITAL

GROUND PLAN

The ward floors are of polished teak, and almost the whole of the rest of the ground floor is laid down with terrazzo. The baths, closets, and sinks are of glazed stoneware of the latest hospital designs. There are special means of ventilation, and the wards are warmed by open fire-places. The elevations are decidedly picturesque, and the inhabitants of Ramsey are to be congratulated on their good fortune on having obtained such a handsome and useful gift from the trustees of the Henry Bloom Noble Estate. We regret that the cost of the hospital is not stated, as it would have been interesting to know what a good cottage hospital can be built for in the present day. The architects were Messrs. Henman and Cooper, of Birmingham; the contractors were Messrs. Callow and Sons, of Ramsey; and Mr. Wattleworth acted as clerk of the works.

## Figures and Tables

**Figure f1:**